# Identification and characterization of the causative triatomine bugs of anaphylactic shock in Zhanjiang, China

**DOI:** 10.1186/s40249-018-0509-1

**Published:** 2018-12-11

**Authors:** Ya-Lan Huang, Da-Na Huang, Wei-Hua Wu, Fan Yang, Xiao-Min Zhang, Miao Wang, Yi-Jun Tang, Qian Zhang, Li-Fei Peng, Ren-Li Zhang

**Affiliations:** 1 Shenzhen Center for Disease Control and Prevention, Shenzhen, 518055 China; 2 Department of Parasitology, Guangdong Medical University, Zhanjiang, 524005 China

**Keywords:** *Triatoma rubrofasciata*, Chagas disease, *Trypanosoma cruzi*, Anaphylactic shock

## Abstract

**Background:**

Two health concerns primarily related to triatomine bugs are transmission of *Trypanosoma cruzi* through infective feces, and allergic reactions induced by triatomine bites. In the Southwestern United States, reduviid bugs bites commonly cause insect allergy. In South China, four cases of anaphylactic shock have been reported after this bite exposure. To further classify the species of these bugs and confirm the sensitization of the triatomine saliva, we caught triatomine bugs from the region where the bites occurred and performed phylogenetic and immunohistochemical (IHC) analysis.

**Methods:**

Triatomine bugs were collected in Donghai Island of Zhanjiang City in South China. The genomic DNA was extracted from three legs of the bugs. The fragments of mitochondrial 16S rRNA, cytochrome c oxidase subunit I (COI) gene and nuclear ribosomal 18S and 28S rRNA genes were obtained by PCR and sequenced. A phylogenetic tree was constructed based on the sequence of 16S rRNA gene using a maximum likelihood method with MEGA 7.0 software. Trypanosomal specific fragments and vertebrate COI genes were amplified from the fecal DNA to detect the infection of trypanosomes and analyze the blood feeding patterns, respectively. Paraffin-embedded sections were then prepared from adult triatomines and sent for IHC staining.

**Results:**

We collected two adult triatomine bugs in Donghai Island. Morphological and molecular analyses indicated that the triatomines were *Triatoma rubrofasciata.* No fragments of *T. cruzi* or other trypanosomes were detected from the fecal DNA. Mitochondrial gene segments of *Homo sapiens* and *Mus musculus* were successfully amplified. The allergens which induced specific IgE antibodies in human serum were localized in the triatomine saliva by IHC assay.

**Conclusions:**

The two triatomine bugs from Donghai Island were *T. rubrofasciata*. They had bitten humans and mice. Their saliva should contain the allergens related to the allergic symptoms and even anaphylactic shock of exposed residents. Great consideration should be given to this triatomine bugs due to their considerable distribution and potential threat to public health in South China.

## Background

According to the World Health Organization (WHO), vector borne diseases account for more than 17% of all infectious disease and cause more than 700 000 deaths annually [1]. Most of them are transmitted by bloodsucking insects such as mosquitoes, sandflies, ticks, flies and triatomine bugs. The reduviid subfamily Triatominae (triatomine bugs) is a group of medically important insects characterized by obligate hematophagy and transmitting *Trypanosoma cruzi* [2]. These parasites can invade the human body after the individual contacts with the feces of an infected triatomine bug, causing a potentially life-threatening illness called Chagas disease [3].

As one of 21 subfamilies in the large family Reduviidae, Triatominae consists of 5 tribes, 15 genera and 151 species at present [4]. Most triatomine species are reported to be distributed in the Americas roughly from 46°N to 46°S, except for the aberrant Indian genus *Linshcoteus* and the tropicoplitan *Triatoma rubrofasciata* [5, 6]. Triatomines inhabited in Asia contained six species in the genus *Linshcoteus* and eight species in the genus *Triatoma* [7, 8]. In China, two species of triatomines have been recorded: *T. sinica* Hsaio collected in Nanjing in 1965, and *T. rubrofasciata*, with a wider distribution in South China including Guangdong, Guangxi, Hainan and Taiwan [8, 9].

In addition, triatomine bites can cause allergic reactions in humans, which pose another great threat to public health in the endemic regions [10]. Anaphylaxis is a rapidly developing and life-threatening hypersensitive reaction caused by repeated exposure to a specific allergen such as drugs, foods, or insect stings [11]. Although anaphylaxis caused by triatomine bites was reported early in 1894, it is still an unrecognized problem easily ignored by patients and medical staffs because this painless sting usually happens in the evening [10]. The manifestations of allergic reactions to triatomine bites vary from localized allergies to more systematic reactions such as anaphylactic shock and even death [12]. Anaphylactic reactions to triatomines were mainly reported in the endemic areas such as Western and Southwestern United States [13]. Several species (*T. protracta, T. gerstaeckeri, T. sanguisuga, T. rubida, T. recurve, Paratriatoma hirsuta and T. rubrofasciata*, etc) of *Triatoma* genera have been suggested to be associated with allergic reactions in the United States [10].

In China, few studies have been performed on triatomine bugs except for sporadic case reports. Dermatitis caused by *T. rubrofasciata* bites was recorded in Hainan in 1986 [9]. From 2000 to 2003, four local residents developed anaphylactic shock after triatomine bites on Donghai Island of Zhanjiang City in South China [14]. They displayed insect bite wounds and developed typical allergic symptoms, such as skin rashes, chest tightness, dyspnea and circulatory compromises. Anti-shock treatment achieved good responses and reversed the life-threatening conditions. In this study, to identify the classification of the involved triatomines and investigate the sensitization of the triatomine saliva, as well as to determine the infection of *T. cruzi* and characterize the blood feeding pattern, we searched the beds, walls and roof cracks of the houses and caught two triatomines from Donghai Island and collected their feces for further analysis.

## Materials and methods

### Triatomine and human serum collection

**Fig. 1:**
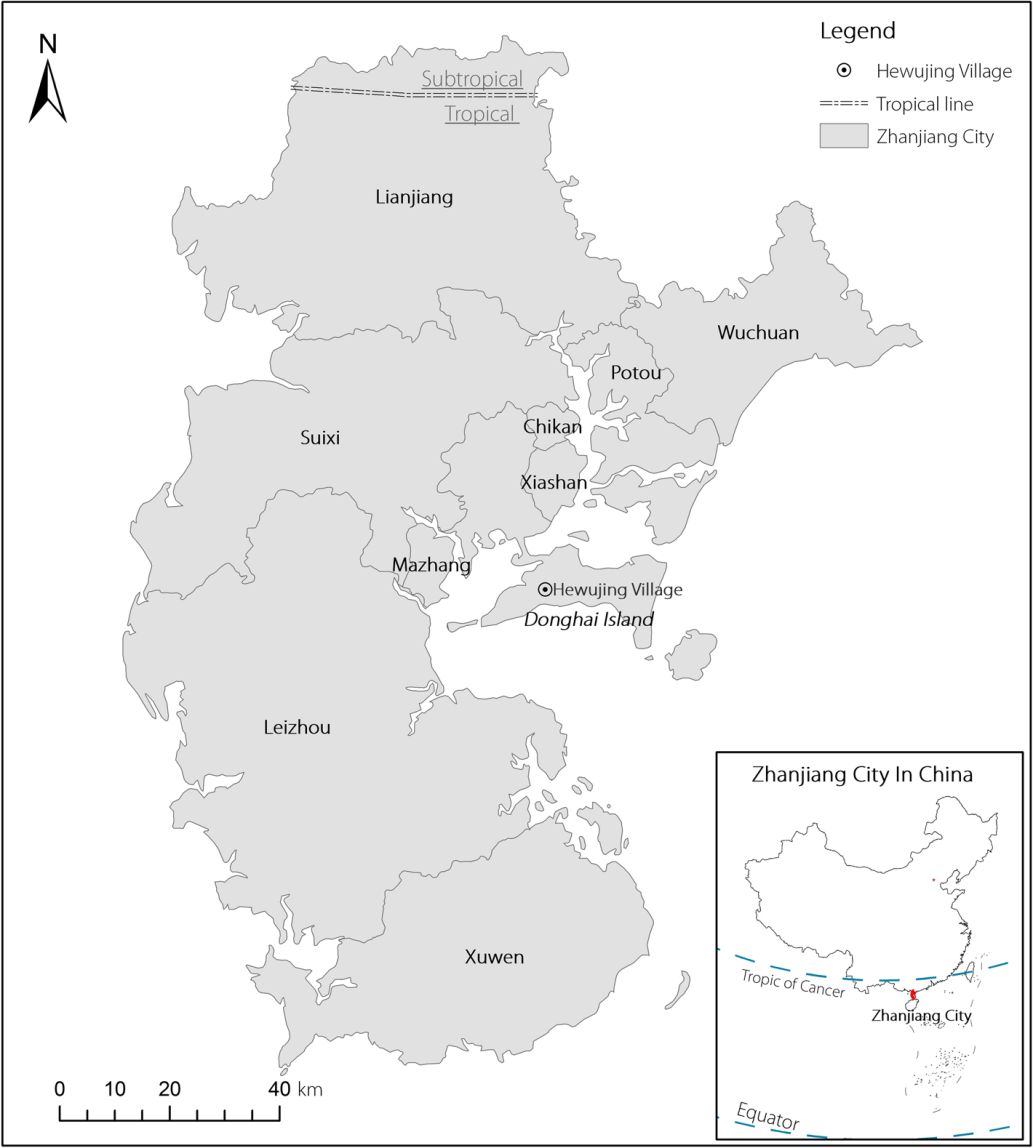
The location of Hewujing Village on Donghai Island of South China

The triatomine bugs were caught from Hewujing Village, Min’an Town of Donghai Island in July, 2017 (Fig. 1). In this region, four local residents have been reported to be bitten by the triatomine bugs and showed anaphylactic shock. The beds, wall and roof cracks of villagers’ houses were the primary collection sites. The housekeeper who was bitten by triatomines and developed allergic reactions was also included in the research. We collected the serum of the allergic person for immunohistochemistry (IHC) analysis. Serum of the healthy donor in Shenzhen City who never contacted with triatomine bugs was also collected.

### Genomic DNA extraction, sequencing and phylogenetic analysis

Total DNA was extracted from three legs of two bugs with QIAamp DNA Mini Kit (Qiagen 51 304, Germany) combined with the TissueLyser LT (Qiagen 85 600, Germany) according to the manufacturer’s instructions. The primers that were used to amplify mitochondrial 16S rRNA, cytochrome c oxidase subunit I (COI) gene and nuclear ribosomal 18S and 28S rRNA gene are listed in Table 1 [15–17]. PCR reactions were conducted with an AccuPrime™ Taq DNA Polymerase System (Invitrogen 12 339 016, USA). The PCR products were examined with 1% agarose gel electrophoresis, purified with a QIAquick PCR Purification Kit (Qiagen 28 104, Germany) and sequenced with Sanger sequencing technology (BGI, Shenzhen, China). The obtained sequences were assembled using SeqMan Lasergene v. 7.0 software (DNAStar, Inc., Wisconsin, USA) and submitted to GenBank (https://www.ncbi.nlm.nih.gov/genbank/) under the accession numbers MG674717, MG923959, MG674716 and MG675575. A phylogenetic tree was constructed based on the sequence of 16 s rRNA genes using the maximum likelihood method with MEGA 7.0 software (https://www.megasoftware.net/download_form). Evolutionary distances were calculated using the Tamura-Nei substitution model and the bootstrap consensus tree inferred from 1000 replicates.

**Table 1:** Primers used to amplify the mitochondrial 16S rRNA, cytochrome c oxidase subunit I (COI) gene and nuclear ribosomal 18S and 28S rRNA genes

Gene target	Forward primer (5’→3’)	Reverse primer (5’→3’)	Expected length (bp)
16S rRNA	CGCCTGTTTATCAAAAACAT [15]	CTCCGGTTTGAACTCAGATCA [15]	552
28S rRNA	GCGAGTCGTGTTGCTTGATAGTGCAG [16]	TTGGTCCGTGTTTCAAGACGGG [16]	710
COI	CCTGCAGGAGGAGGAGAYCC [17]	TAAGCGTCTGGGTAGTCTGARTAKCG [17]	650
18S rRNA-1^a^	TGGTTGATCCTGCCAGTAGTC	TCGACACTCGTTTAAGAGCACC	822
18S rRNA-2^a^	TGTTGCGGTTAAAAAGCTCG	TCGGAATTAACCAGACAAATCG	803
18S rRNA-3^a^	AGGTTCGAAGGCGATCAGATAC	TCCTTCCGCAGGTTCACCTA	829

^a^designed for this study

### Fecal DNA extraction and blood feeding patterns of the two triatomine bugs

The feces of adult bugs before laboratory rearing were collected for DNA extraction with QIAamp®DNA Stool Mini Kit (Cat. No. 51504, QIAGEN, Hilden, Germany). To investigate whether the triatomine bugs had sucked blood of vertebrates in natural environment, the vertebrate cytochrome c oxidase I subunit gene (vCOI) was amplified with a set of the primers (vCOI_F:5’-AAGAATCAGAATARGTGTTG-3′; vCOI_R: 5’-AACCACAAAGACATTGGCAC-3′) from fecal DNA samples according to previous study [18]. The purified PCR fragments were inserted into pGEM-T Easy cloning vectors (Promega A1360, USA) and sent to be sequenced (BGI, Shenzhen, China). The sequenced gene fragments were compared with those from GenBank to identify the blood meal sources.

### Identification of trypanosomes by nest-PCR and q-PCR

Five microliters of fecal DNA was used for PCR amplification in a 50 μl reaction with an AccuPrime™ *Taq* DNA Polymerase System (Cat. No. 12339016, Invitrogen, Carlsbad, CA, USA), as follows: 94 °C for 2 min; 35 cycles at 94 °C for 30 s, 55 °C for 30 s, 68 °C for 1 min. Primers were designed as described previously, targeting the 24S alpha subunit rRNA gene of trypanosomatids and a nested-PCR was subsequently conducted to amplify the *T. cruzi*-specific region of the same gene using primers D71 and D72 [19, 20]. PCR products were examined with 2% agarose gel electrophoresis. Additionally, the qPCR reactions were used to detect *T. cruzi* satellite DNA gene fragments with 2 × *Premix Ex Taq*™ (Code No. RR390A, Takara Bio Inc., Japan) as described previously [21]. Primers and probes were synthesized by Sangon Biotech (Shanghai, China).

### Immunolocalization of salivary allergens in the triatomine bugs

The bodies of adult bugs were fixed with 4% paraformaldehyde. The fixed tissues were embedded in paraffin wax, and processed to obtain 5-μm-pore-size sections. After deparaffinization and rehydration, the slides were steamed in 0.01 mol/L sodium citrate buffer (pH 6.0) for 10 min with the microwave antigen retrieval method. The endogenous peroxidase was quenched with 3% H_2_O_2_ in 80% methanol for 20 min, and then nonspecific absorption was minimized by incubating the sections in 5% normal goat serum in PBS for 30 min at room temperature. The sections were then incubated overnight with a 1:20 dilution of the serum from the allergic patient or with control serum of healthy people. Specific labeling was detected with a horseradish peroxidase (HRP) labeled goat anti-human IgE antibody (ab73901, Abcam, USA) and the DAB Immunohistochemistry Color Development Kit (E670033, Sangon Biotech, Shanghai, China).

## Results

### Morphological characteristics of the triatomine bugs

**Fig. 2:**
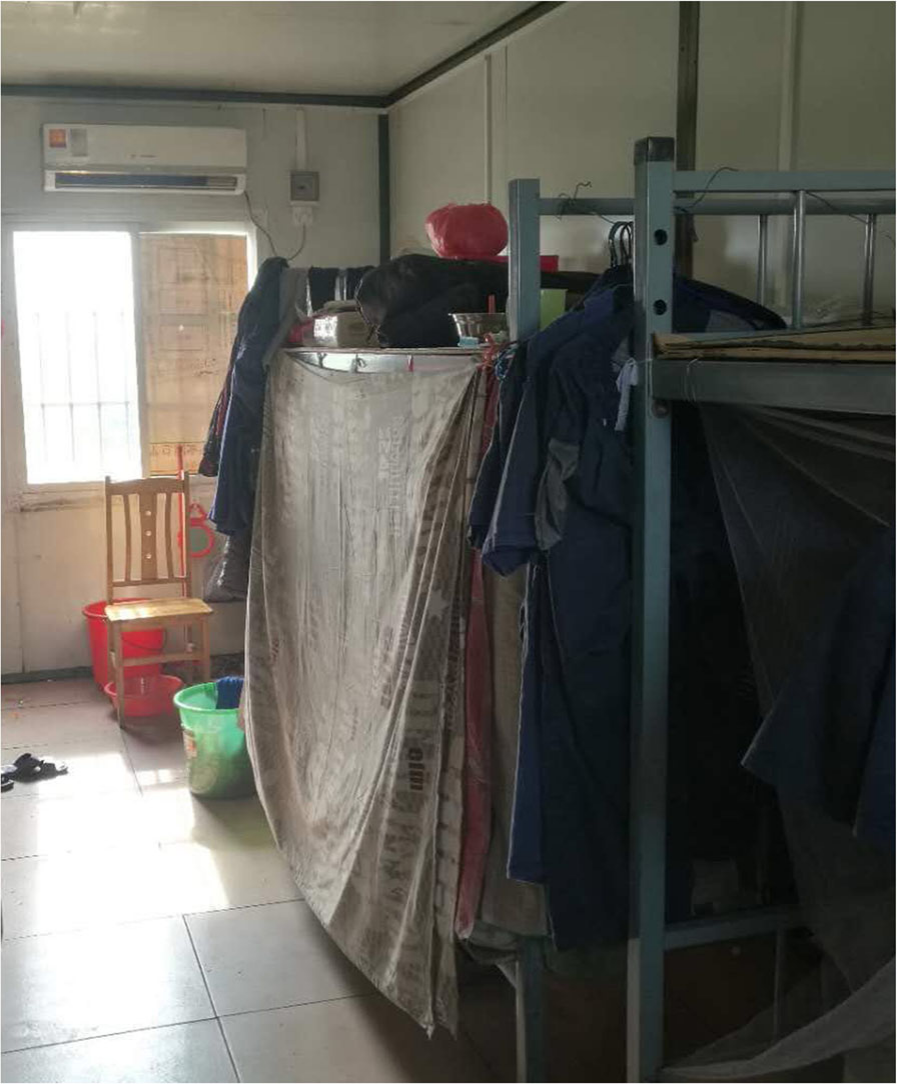
The house where we collected the two adult triatomine bugs

One pair of adult bugs was caught under the bed in a farmer’s house in Hewujing Village, Min’an Town of Donghai Island. The house was crowded and heaped with sundry, clothes and living supplies (Fig. 2). The housekeeper complaint that he had been bitten by this kind of bugs several times and experienced symptoms such as skin rashes, pruritus, sudden increase of heart rate and dyspnea.

**Fig. 3:**
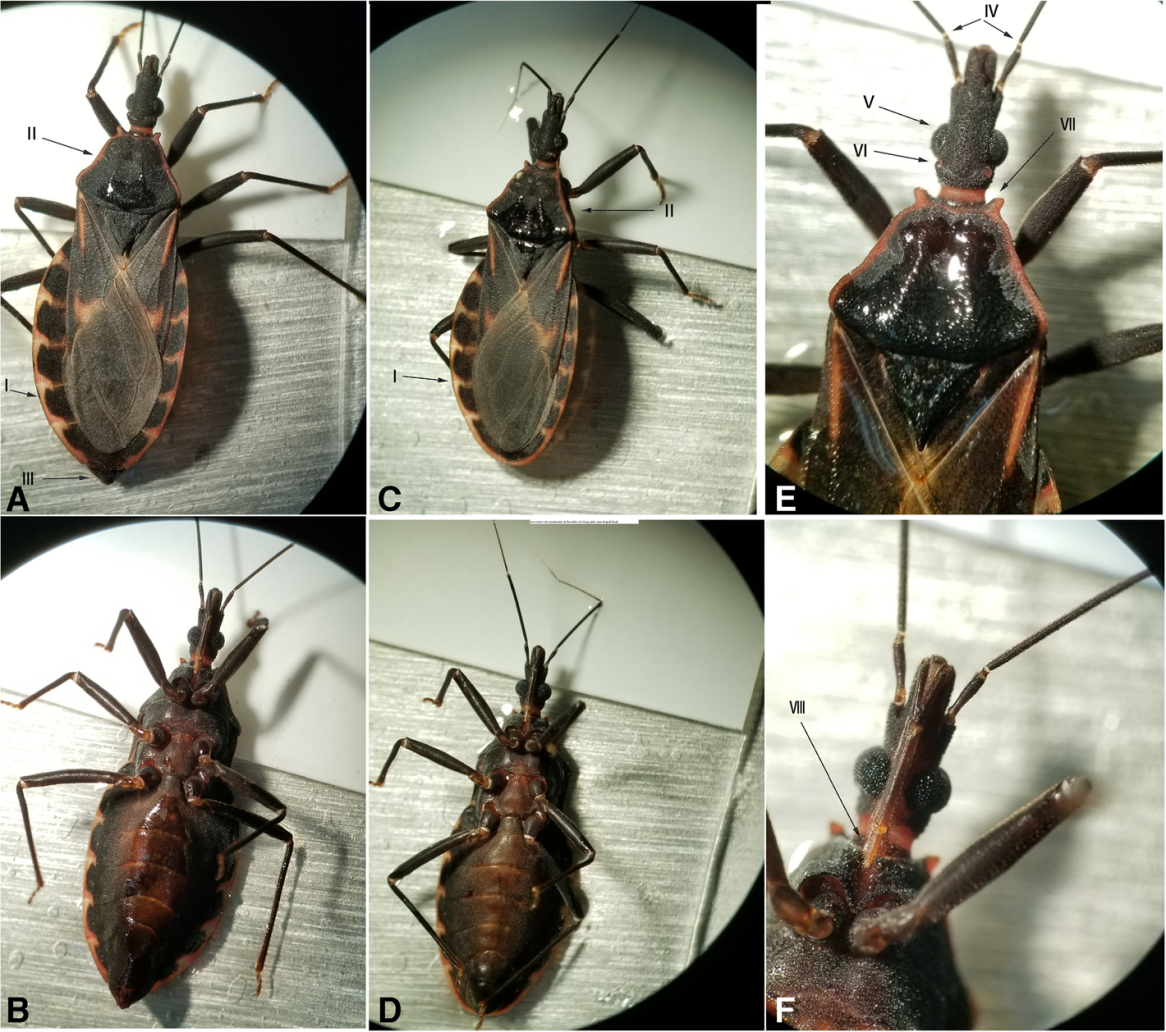
**a** and **b** Dorsal and ventral views of the female *T. rubrofasciata*; **c** and **d** Dorsal and ventral views of the male *T. rubrofasciata*. I: Orange-red margin along the outer edge of the abdomen which extended horizontally between segments; II: Orange-red margin along the side of the pronotum; III: The genital region of the female adult bug was strongly projecting posteriad; IV: The first segment surpassed the apex of its head; V: Eyes at the sides of a long and cone-shaped head; VI: Ocelli; VII: Anterior angles produced into short spines of a reddish yellow color; VIII: The stout proboscis hinged beneath the thorax, covered with short hairs which were progressively longer towards tip

The two bugs were identified to be heterosexual and showed morphological characteristics of *T. rubrofasciata* (Fig. 3). The female and male were approximately 25 mm and 20 mm in length. The genital region of the female adult bug was strongly projecting posteriad (Fig. 3a and b). Adults exhibited orange-red margin along the side of the pronotum and the outer edge of the abdomen which extended horizontally between segments (Fig. 3a, b, c and d). The eyes were very prominent at the sides of a long and cone-shaped head (Fig. 3e). The ocelli were behind and above the eyes (Fig. 3e). The antennae had four segments and the first segment surpassed the apex of its head (Fig. 3e). The pronotum was black and sulcated, with two anterior angles produced into short spines of a reddish yellow color (Fig. 3e). The scutellum was dark and broad, triangular to tip (Fig. 3e). The proboscis was stout and hinged beneath the head, and covered with short hairs which were progressively longer towards tip (Fig. 3f).

### Molecular characteristics of the triatomine bugs

In addition to the morphological classification method, molecular and phylogenic analyses were also performed to identify their species. The partial sequences of mitochondrial 16S rRNA (546 bp), COI gene (651 bp), nuclear ribosomal 18S rRNA (1880 bp) and 28S rRNA (693 bp) genes were sequenced and submitted to the GenBank under the accession numbers of MG674717, MG923959, MG674716 and MG675575, respectively. The nucleotide sequences of these DNA markers from the female and male triatomine bugs were absolutely the same. These fragments showed more than 98% identity with the genes of *T. rubrofasciata* identified and sequenced in other areas such as Taiwan of China, Vietnam and Brazil.

**Fig. 4:**
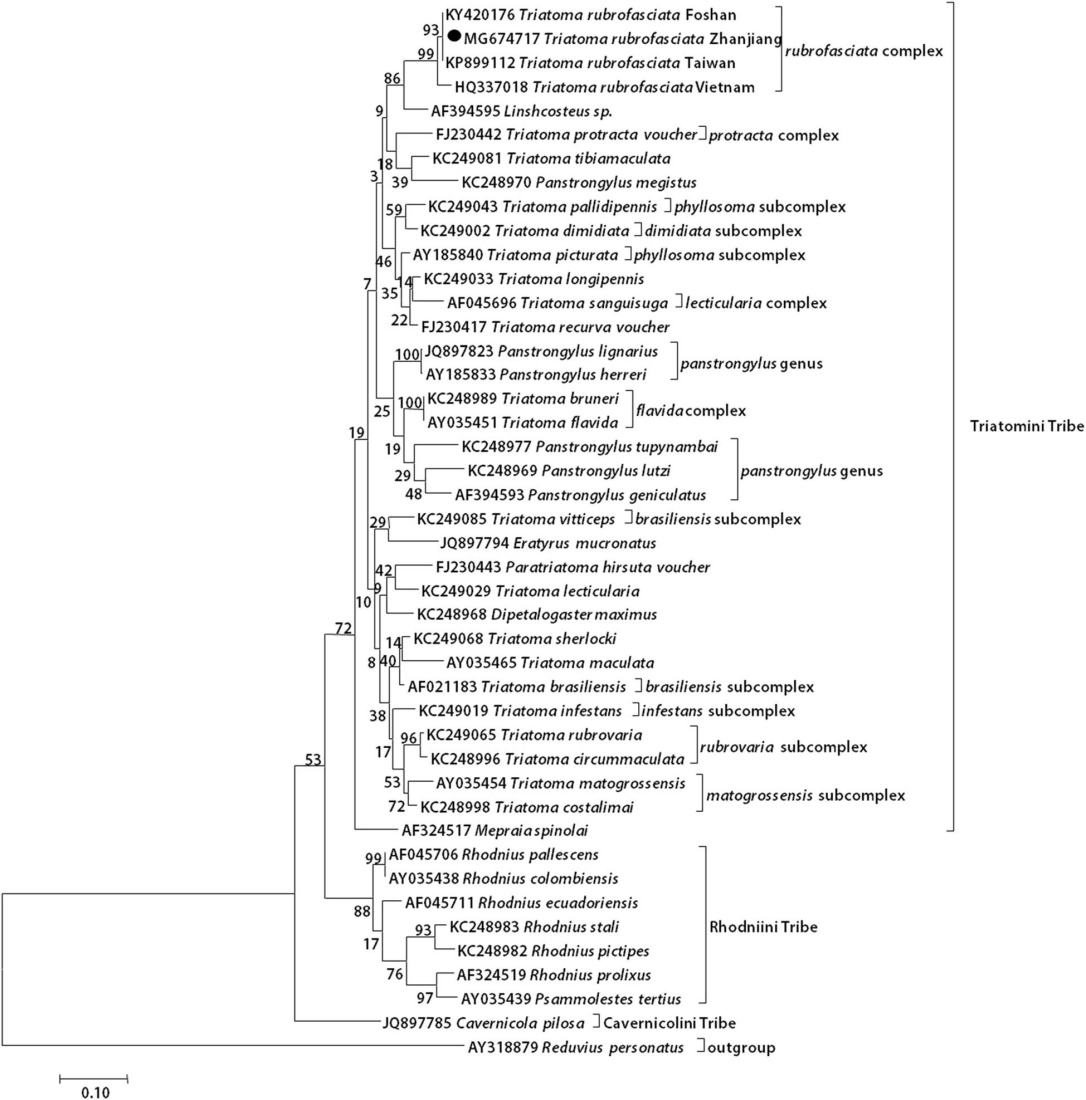
Phylogenetic tree inferred by mitochondrial 16S rRNA genes. The Maximum Likelihood method with the Tamura-Nei mode was used

A phylogenetic tree was established based on the triatomine 16S rRNA gene of Zhanjiang bugs and other reference sequences in GenBank (Fig. 4). According to the phylogenetic tree, the Triatomini tribe was divided into three main clades: (1) Northern American *Triatoma* (*dimidiata* subcomplex + *protracta* complex + *lecticularia* subcomplex + *phyllosoma* subcomplex) + *T. rubrofasciata* complex +*Linshcosteus*; (2) *Panstrongylus* + *flavida* complex; (3) Southern American *Triatoma* (including *infestans*, *brasiliensis*, *rubrovaria* and *matogrossensis* subcomplex as well as *Mepraia* and *Eratyrus*). The 16S rRNA sequence of the Zhanjiang triatomine was identical to that of Taiwan and Foshan. This Chinese group was closest to the Vietnam strain (HQ337018). The Chinese triatomines and Vietnam strain were classified in the *rubrofasciata* complex of the Northern American *Triatoma* clade.

### Identification of blood feeding patterns and detection of trypanosomes in feces of bugs

The mitochondrial COI genes of both *Homo sapiens* and *Mus musculus* were detected in the feces samples of the field caught triatomines, suggesting that these triatomines had bitten humans and mice.

The gene fragments of trypanosomatids and *T. cruzi* could not be amplified from the fecal DNA samples of the field-caught triatomines. It suggested that the two *T. rubrofasciata* from Donghai Island did not host the trypanosome parasites.

### Immunolocalization of salivary allergens in the triatomine bugs

**Fig. 5:**
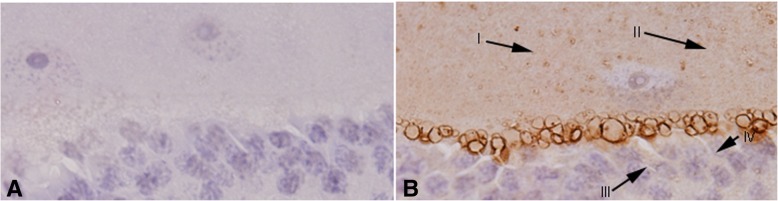
Immunolocalization of salivary allergens of the triatomine bug. The bugs were fixed, embedded, and sectioned as described in *Materials and Methods*. **a** The serum (1:20) of healthy people as control antibody; **b** The serum (1:20) of the allergic patient as primary antibody. HRP labeled secondary antibody (goat anti-human IgE antibody) localizes allergens in the luminal contents of the salivary glands (I and II) and the cytoplasm of epithelium (III and IV) (original magnification, × 400)

IHC was performed to identify whether the patient could produce specific IgE antibodies in the serum after the stimulation of the triatomine saliva proteins. The goat anti-human IgE antibody was used as the secondary antibody. We found that the salivary glands of the field-caught triatomines that were incubated with the serum of allergic patient showed brown yellow color. The positive staining was localized in the cytoplasm of epithelium and the luminal contents of the salivary glands (Fig. 5b). However, the salivary glands that were incubated with the serum of healthy people as primary antibody showed negative staining (Fig. 5a). The results suggested that there were IgE antibodies specific to the triatomine saliva in the serum of the allergic patient.

## Discussion

*T. rubrofasciata* is the most widespread species in Asia and distributed throughout the Indo-Pacific region including Andaman Islands, Borneo, Burma, Cambodia, Carolina Islands, China, Comoros Islands, India, Indonesia, Japan, Madagascar, Mauritius, Philippines, Reunion, Rodriguez Islands, Sri Lanka, Singapore, Seychelles, Thailand, Tonga, Vietnam [7–9, 22, 23]. We caught two triatomine bugs from Donghai Island of Zhanjiang in South China from a farmer’s house who had been bitten by the bugs for several times and developed allergic symptoms. The morphological and phylogenetic analyses confirmed that these two bugs were *T. rubrofasciata.* The fecal examination suggested that the bugs had sucked blood from humans and mice. Therefore, the local residents would be under the threat of infectious disease and bite related complications caused by *T. rubrofasciata*.

Donghai Island (E 110°11′–110°31′, N 20°55′–21°05′) is located in the south of the equator and near Pacific Ocean and Southeast Asian countries. It has a tropical monsoon climate and abundant rainfall suitable for the breeding of a variety of hematophagous insects. The tropical agriculture is well developed and vegetation is rich which can provide appropriate peri-domestic shelters for triatomines. The house where we found the triatomine bugs was cluttered with living supplies, which provided the bugs good domestic shelters. Klotz et al. thought after the entry of the triatomine bugs into houses they may commonly feed on house owners and pets for months or until discovered [24]. Thus, it is very important to launch a health education about the triatomine habitats to the local residents to reduce the risk of exposure to these dangerous bugs.

All species of triatomines need the blood of a vertebrate for their complete development [25]. All triatomines defecate shortly after their blood meals [25]. The vertebrate blood genes in the triatomine feces are concordant with its food sources. We detected specific mitochondrial COI gene of both *Homo sapiens* and *Mus musculus* from their feces samples, which hinted that these two bugs had bitten both humans and mice.

All species of triatomines are considered capable of transmitting *T. cruzi* including *T. rubrofasciata* through their infective feces [6]. In addition, *T. rubrofasciata* can transmit another species of trypanosome-*T. conorhini,* which was found to infect *Rattus rattus* and *Macaca cyclopis* [7, 22, 26]. *T. conorhini* infections in humans have not been reported, but it may still be a threat to the immunocompromised patients [7]. According to our results, no trypanosomes including *T. cruzi* and *T. conorhini* were detected in the feces of the wild *T. rubrofasciata*. There is also no record of Chagas disease in China so far [27]. Chagas disease was once distributed only in the Americas, principally Latin America, but later began to spread to other continents [3]. The migration of insect vectors might increase the risk of disease spreading. As a result, it is very necessary to monitor this important insect and prevent imported Chagas disease.

Apart from the important infectious Chagas disease, the allergic reaction is another health issue caused by the triatomine bites in sensitized individuals [8–12]. In the United States, anaphylaxis is the most serious complication of a triatomine bug bite [12]. A case review indicated that there were at least 110 persons who had allergic reactions to triatomine bites in Mariposa County of California alone [28]. In Southeast Asia, the bite reaction is also the main nuisance posed by triatomine bugs [8]. Cases of allergic reactions to the bites of *T. rubrofasciata* have been reported in Singapore, Philippines, Vietnam and China [8, 9, 14, 23, 29]. But up to now, the pathogenesis of this allergy has not received systematical studies in the whole Asia area. In the Americas, the researchers mainly focused on the allergies to *T. protracta* [30–33]. We firstly collected the serum of allergic patient and investigated the sensitization of the *T. rubrofasciata* saliva.

During the triatomine’s blood suckling, the triatomine saliva will travel through the salivary ducts to the proboscis and be injected into the bite victims [30]. The allergens responsible for sensitization of *T. protracta* were found in the saliva [30]. It has been proven that the saliva induced IgE-mediated hypersensitivity is the cause of the triatomine bite related anaphylaxis [31, 32]. We found that the IgE antibodies specific to *T. rubrofasciata* saliva were present in the serum of the allergic patient who had been bitten for many times through the IHC analysis. Similarly, Rohr et al. also measured that the IgE levels specific to *T. protracta* salivary extracts in the sera of five patients with life threatening allergic reactions were 200 to 400% greater than that of the control sera by the radioimmunoassay method [33]. But the particular components in the saliva responsible for the allergies are remained to be determined. Previous studies indicate that major allergic proteins of *T. protracta* are of low molecular weight (18000–20 000 Daltons) such as procalin [12, 30]. In addition, a study demonstrates that the salivary allergens do not cross react between different triatomine species [30]. There were no reports and literature of relevant studies on the specific allergen proteins of *T. rubrofasciata* to date. With specific IgE antibodies in the serum of the allergic patient, we can identify and construct the allergens in salivary proteins of *T. rubrofasciata* once collected enough bugs. This study was conducted to determine the presence of trypanosome infections in local triatomine bugs and the sensitization of the *T. rubrofasciata*. Due to the limited sample size and sampling area, it could not cover all the conditions of Chinese triatomines. With the established methods, we would expand the study area and deepen the research on mechanisms of anaphylaxis.

## Conclusions

*T. rubrofasciata* was the causative triatomine responsible for the anaphylactic shock experienced by the residents in Donghai Island of Zhanjiang City in South China. The wild caught bugs had sucked blood from humans and mice. No evidence was found for the infections of trypanosomes in these triatomine bugs. Our findings revealed the basic characteristics of the *T. rubrofasciata* in South China and their ability to cause anaphylaxis, which may help you to have a better understanding of this dangerous bug and benefit for its control. Further identification of the specific allergens in the saliva is warranted.

## Abbreviations

COI: Cytochrome c oxidase subunit I
HRP: Horseradish peroxidase
IHC: Immunohistochemistry
WHO: World Health Organization
